# Accelerated diastolic dysfunction in premenopausal women with rheumatoid arthritis

**DOI:** 10.1186/s13075-021-02629-1

**Published:** 2021-09-24

**Authors:** Gee Hee Kim, Yune-Jung Park

**Affiliations:** 1 Division of Cardiology, Department of Internal Medicine, St. Vincent’s Hospital, College of Medicine, The Catholic University of Korea, Suwon, Korea; 2 Division of Rheumatology, Department of Internal Medicine, St. Vincent’s Hospital, College of Medicine, The Catholic University of Korea, Suwon, Korea

**Keywords:** Diastolic dysfunction, Rheumatoid arthritis, Premenopausal women

## Abstract

**Background:**

Disturbances of diastolic function precede systolic heart failure and, although clinically silent, represent the earliest sign of cardiac involvement. Diastolic dysfunction (DD) is associated with age, gender (female), and hypertension. However, little is known about the age-specific incidence rates and risk factors for DD in patients with rheumatoid arthritis (RA).

**Methods:**

We used standard two-dimensional/Doppler echocardiography to screen for the presence of diastolic dysfunction in 61 patients with RA and 107 healthy subjects. All participants were premenopausal women with no history of hypertension. DD includes an impaired relaxation with or without increased left ventricular (LV) filling pressures, pseudonormal filling, and restrictive filling based on parameters measured using echocardiography.

**Results:**

The two groups were similar with respect to age (*P*=0.269). Patients with RA had significantly higher LV mass index, LV filling pressure, and lower E/A velocity than controls. All patients had preserved ejection fraction (EF ≥50%). DD was more common in patients with RA at 47% compared to 26% in the controls (*P*=0.004). Women with RA in the 30- to 49-year age range were over 3.5 times more likely to have DD than those of similar age in the control group (OR=3.54; 95% CI 1.27 to 9.85). Among patients with RA, high CRP levels were independently associated with DD even after adjustment for cardiovascular risk factors (*P*=0.009).

**Conclusions:**

In premenopausal women with RA, DD is much more common and the age of onset is reduced. Early screening of myocardial function may provide an opportunity for preventing future cardiovascular disease.

**Supplementary Information:**

The online version contains supplementary material available at 10.1186/s13075-021-02629-1.

## Introduction

As compared to the general population, patients with rheumatoid arthritis (RA) experience a higher incidence of heart failure (HF) [1]. HF is a major risk factor for mortality in RA and is associated with cardiovascular deaths in patients with RA [2]. Various studies have shown that subclinical left ventricular (LV) diastolic dysfunction predicts future development of clinical HF [3, 4].

Diastolic dysfunction refers to abnormal mechanical properties of the myocardium and includes impaired LV diastolic distensibility, abnormal filling, and slow or delayed relaxation [5]. According to a recent systematic review, diastolic dysfunction affects approximately 36% of the population older than 60 years [6] and is closely associated with several cardiovascular risk factors, including hypertension, obesity, and diabetes [7, 8]. In the Olmsted County Heart Function Study (a population-based cohort), 4 years of follow-up revealed that LV diastolic dysfunction is highly prevalent, tends to worsen over time, and is associated with advancing age and development of HF during 6 years of subsequent follow-up [9].

Previous reports have demonstrated that the prevalence of diastolic dysfunction is increased in patients with RA [10]. Although inflammation such as tumor necrosis factor-alpha (TNF-α) and interleukin-6 (IL-6) levels, cardiotoxic medication, and RA disease itself have been thought to be the risk factors for the development of diastolic impairment, the precise mechanisms involved in increased cardiovascular disease (CVD) risk are various and remain elusive [11]. Moreover, few studies have been conducted specifically investigating age, which is one of the strongest risk factors. In this study, we investigate age-related prevalence and risk factors of diastolic dysfunction in RA patients.

## Methods

### Patients

We included patients age ≥18 years with adult-onset RA (*n*=61), classified by the American College of Rheumatology (ACR)/European League Against Rheumatism (EULAR) 2010 criteria [12] who had received treatment for at least 6 consecutive months. The status of menopause was confirmed through the questionnaire and premenopausal women were enrolled. Age- and sex-matched individuals (*n*=107) who underwent health checkup during the same period were included as a control group.

All subjects underwent a clinical and laboratory evaluation, and the following information was collected: traditional CVD risk factors, such as blood pressure, dyslipidemia, diabetes mellitus, smoking status, and body mass index (BMI). The status of cigarette smoking and menopause were elicited by a self-administered questionnaire and current smoking was defined as any smoking within the past year. Subjects with any of the following conditions were excluded: preexisting overt coronary artery disease, transient ischemic attack, stroke, congestive heart failure, hypertension, renal failure (defined as a serum creatinine level > 3.0 mg/dl), active infection, and pregnancy. Among the CVD risk factors hypertension was excluded because hypertensive patients often already have myocardial changes and the effects of antihypertensive drugs on diastolic function can be different.

Laboratory parameters included complete blood count, blood levels of glucose, creatinine, electrolytes, total cholesterol, low-density lipoprotein cholesterol, high-density lipoprotein (HDL) cholesterol, triglycerides, erythrocyte sedimentation rate (ESR), and C-reactive protein (CRP). Characteristics of disease duration and the presence of extra-articular manifestations, rheumatoid factor (RF), and anti-cyclic citrullinated peptide (Anti-CCP) antibody were recorded for patients with RA. Information about the use of medications including prednisolone, non-steroidal anti-inflammatory drugs (NSAIDs), methotrexate, leflunomide, hydroxychloroquine, sulfasalazine, TNF inhibitors, and statin was retrieved from medical records. Disease activity was evaluated by using the 28-joint assessment (DAS28) score [13]. All subjects gave written consent before entering the study, which was approved by the institutional review board of the Catholic University of Korea (No. VC14OISI0185)

### Transthoracic Doppler echocardiography

Two-dimensional, M-mode, pulsed Doppler and tissue Doppler echocardiography were performed using a Vivid Seven ultrasound machine (GE Medical Systems, Horten, Norway) with a 2.5-MHz transducer. Standard two-dimensional measurements (LV diastolic and systolic dimension, ventricular septum and posterior wall thickness, and left atrial volume) were obtained as recommended by the American Society of Echocardiography [14]. From the apical window, a 1- to 2-mm pulsed Doppler sample volume was placed at the mitral valve tip, and mitral flow velocities from five to 10 cardiac cycles were recorded. The mitral inflow velocities were traced and the following variables were obtained: peak velocity of early diastolic mitral inflow (E), late diastolic mitral inflow (A), and deceleration time of the E velocity [15]. Stroke volume was measured from the LV outflow tract diameter and the pulse-wave Doppler signal. Mitral annular velocities were measured by Doppler tissue imaging using the pulsed-wave mode. The filter was set to exclude high-frequency signals, and the Nyquist limit was adjusted to a range of 15 to 20 cm/s. Gain and sample volume were minimized to allow a clear tissue signal with minimal background noise. Early diastolic mitral annular (Em), late diastolic (Am), and systolic velocities (Sm) of the mitral annulus were measured from the apical 4-chamber view, with a 2- to 5-mm sample volume placed at the septal corner of the mitral annulus. Diastolic dysfunction is categorized by Doppler echocardiographic findings into the following progression: grade I defined as impaired relaxation with or without mild evidence of increased filling pressures, grade II defined as impaired relaxation associated with moderate elevation of filling pressures or pseudonormal filling, and grade III defined as advanced reduction in compliance restrictive filling [16]. In this study, diastolic dysfunction was categorized as grade I, grade II, or grade III.

### Statistical analysis

Variables with a normal distribution are presented as the mean ± SD. Differences between the mean values were examined by Student’s *t*-test. Variables showing a non-normal distribution were expressed as the median and interquartile range (IQR). Nonparametric data were compared between groups using the Mann-Whitney *U* test. For categorical data, the difference in prevalence was evaluated by the chi-square test first and then if there were any warnings Fisher’s two-tailed exact test was performed. Cumulative probability plots were used to display the diastolic dysfunction, which was based on age in patients with RA.

Multivariate regression analysis was performed to investigate risk factors associated with the diastolic dysfunction in patients with RA. Multivariate models included all covariates with associations from univariate analysis with a *P*-value≤0.20 and potential confounding factors known to influence diastolic dysfunction (Supplementary Table 1). *P* values <0.05 were considered significant. Data analyses were performed using R (http://www.R-project.org and web-r.org) software.

## Results

### Characteristics of study population

The baseline characteristics of the 61 RA patients and 107 healthy controls are shown in Table 1. The mean age was 48.1±7.9 years in RA patients and 47.3±9.4 years in healthy subjects (*P*=0.269). The mean body mass index was lower in RA patients than in non-RA controls but there were no significant differences found in lipid profile, creatinine levels, or percentage of patients with diabetes mellitus between the two groups. RA patients had higher diastolic blood pressure and HDL cholesterol levels than controls (*P*=0.011 and *P*<0.001, respectively). In RA patients, the median disease duration was 6.9 years, and the median DAS28 score was 3.0. Forty-five patients (73.8%) were positive for RF and 54 patients (88.5%) were positive for anti-CCP antibodies. Forty-four patients (72.1%) were taking weekly methotrexate, 22 (36.1%) were on leflunomide, and 16 (26.2%) were on sulfasalazine. Forty-two patients (68.9%) were treated with low-dose prednisolone (≤7.5 mg/day).

**Table 1:** Baseline characteristics of the study participants

	RA patients (*n*=61)	Controls (*n*=107)	***P*** value^**†**^
Age, years	48.1±7.9	47.3±9.4	0.269
Duration of disease, years	6 [3–12]	NA	NA
Current smoker, *n* (%)	5 (8.2)	2 (1.9)	0.100^‡^
Blood pressure, mmHg			
Systolic	125.7±18.1	124.4±16.9	0.075
Diastolic	81.4±11.2	78.8±18.1	0.011
Diabetes mellitus, *n* (%)	1 (0.02)	4 (0.04)	0.654^‡^
Body mass index, kg/m^2^	22.8±3.5	23.6±3.2	0.315
Creatinine, mg/dl	0.62±0.11	0.69±0.11	0.121
Cholesterol, mg/dl			
Total	185.2±31.9	193.1±44.0	0.355
High-density lipoprotein	57.9±16.4	46.6±12.3	<0.001
Low-density lipoprotein	94.9±25.4	113.9±35.5	0.068
Triglyceride, mg/dl	93.0±48.8	117.3±83.4	0.425
Statin, *n* (%)	7 (11.5)	0 (0)	<0.001^‡^

Data are presented as mean ± standard deviation, median [interquartile range], or number (percentage) as appropriate

*NA* not applicable

^†^Unless otherwise noted, two-sample *t* test was used

^‡^Fisher’s two-tailed exact test was used

### Prevalence of diastolic dysfunction and echocardiography findings

Table 2 shows echocardiography findings in patients with RA. The incidence of diastolic dysfunction was increased in patients with RA (odds ratio 2.18, 95% confidence interval (CI)1.13–4.20, *P*=0.020). There were significant differences in LVEDD, LV mass index, E/Em, E and A wave velocity, Sm, and Em between the two groups.

**Table 2:** Comparison of echocardiography findings between patients with rheumatoid arthritis (RA) and control subjects

Variables	RA patients (*n*=61)	Controls (*n*=107)	***P value***
**LVEDD, mm**	46 (44.3–49.3)	45.0 (42.6–47.0)	0.033
**LVESD, mm**	29.1 (27.2–31.4)	28.1 (26.0–30.0)	0.131
**LV mass index, g/m**^**2**^	151.7 (128.2–173.1)	140 (119–165)	0.043
**LV ejection fraction, %**	64 (61.7–65.8)	64.6 (62.0–67.3)	0.349
**LV filling pressure, E/Em**	9.4 (8.5–10.1)	9.0 (8.7–10.0)	0.036
**Transmitral E wave velocity, cm/s**	68.5 (55.3–79.9)	72.4 (64.0–82.8)	0.008
**Transmitral A wave velocity, cm/s**	70.0 (57.0–81.8)	65.8 (57.0–77.0)	0.045
**Transmitral E wave / A wave verocity**	0.93 (0.77–1.28)	1.15 (0.85–1.32)	0.003
**Systolic tissue velocity (Sm), cm/s**	6.9 (6.1–8.0)	8.0 (7.0–9.0)	<0.001
**Early diastolic tissue velocity (Em), cm/s**	7.7 (6.0–10.4)	9.8 (7.4–11.9)	0.001
**Late diastolic tissue velocity (Am), cm/s**	8.7 (7.2–9.6)	9.3 (8.0–10.5)	0.105
**Transmitral E wave deceleration time, ms**	201 (175.7–227.3)	190.4 (163.7–214.8)	0.047

Data are presented as median (IQR). *LVEDD*, left ventricular end diastolic dysfunction; *LVESD*, left ventricular end systolic dysfunction; *E*, transmitral E wave velocity; *A*, transmitral A wave velocity; *Sm*, systolic tissue velocity; *Em*, early diastolic tissue velocity; *Am*, late diastolic tissue velocity; *DT*, transmitral E wave deceleration time

### Risk factors associated with diastolic dysfunction in RA patients

Univariate and multivariate regression analyses were applied to identify the risk factors for diastolic dysfunction in patients with RA. In multivariate logistic regression analysis, older age (*P* = 0.035) and CRP levels (*P* = 0.022) were independently associated with diastolic dysfunction in the RA patients (Supplementary Table 1). Elevated serum CRP levels remained significant after adjustment for age (*P*=0.009) (Table 3).

**Table 3:** Characteristics of patients with rheumatoid arthritis, according to the presence or absence of diastolic dysfunction

Characteristics	Diastolic dysfunction		*P*-value^†^	Adjusted for age	*P*-value^‡^
	Presence (*n*=28)	Absence (*n*=33)		Odds ratio^‡^ (95% CI)	
Age, years	50.0 [48.5–53.0]	48.0 [41.0–51.0]	0.025	NA	NA
Disease duration, years	6.5 [2.0–8.5]	6.0 [3.0–11.0]	0.303	0.998 (0.916–1.086)	0.957
Current smoker, *n* (%)	1 (3.6)	4 (12.1)	0.363^∫^	0.286 (0.040–2.037)	0.211
Blood pressure, mmHg					
Systolic	122.0 [117.0–135.5]	125.0 [112.0–131.0]	0.739	0.979 (0.931–1.029)	0.406
Diastolic	80.0 [75.0–89.0]	80.0 [72.0–86.0]	0.249	1.029 (0.962–1.101)	0.407
Diabetes mellitus, *n* (%)	0 (0.0)	1 (3.0)	0.999^∫^	0.000 (0.000–0.000)	0.999
BMI, kg/m^2^	22.9 ± 3.2	22.9 ± 3.4	0.971^∫∫^	0.918 (0.767–1.099)	0.350
Creatinine, mg/dl	0.66 ± 0.12	0.63 ± 0.10	0.464^∫∫^	1.100 (0.007–1.712)	0.171
Cholesterol, mg/dl					
Total	185.5 ± 37.5	182.1 ± 28.6	0.706^∫∫^	1.006 (0.989–1.024)	0.490
HDL	48.0 [40.0–65.0]	53.0 [45.0–64.5]	0.291	1.004 (0.970–1.040)	0.808
LDL	116.2 [85.0–126.4]	106.2[88.0–121.7]	0.468	0.959 (0.899–1.023)	0.200
Triglyceride, mg/dl	94.0[59.0–139.0]	90.5[53.5–109.0]	0.373	0.999 (0.987–1.010)	0.816
ESR, mm/hour	24.5 [13.5–48.5]	24.0 [14.0–39.0]	0.772	1.002 (0.974–1.030)	0.899
CRP, mg/dl	0.9 [ 0.1–1.3]	0.2 [ 0.1–0.5]	0.027	2.077 (1.204–3.585)	0.009
RF, *n* (%) ^¶^	21 (75.0)	24 (72.7)	0.999^∫^	0.999 (0.995–1.002)	0.471
Anti-CCP, *n* (%)^¶^	26 (92.6)	28 (84.8)	0.437^∫^	1.920 (0.483–7.626)	0.354
DAS28	3.0 ± 0.7	3.0 ± 1.0	0.850^∫∫^	0.916 (0.209–4.016)	0.907
Prednisolone, *n* (%)	19 (67.9)	23 (70.0)	0.953^∫^	0.628 (0.196–2.009)	0.433
Methotrexate, *n* (%)	20 (71.4)	24 (72.7)	0.999^∫^	1.228 (0.386–3.911)	0.728
Hydroxychloroquine, *n* (%)	18 (64.3)	17 (51.5)	0.436^∫^	1.361 (0.464–3.991)	0.574
Sulfasalazine, *n* (%)	9 (31.1)	7 (21.2)	0.390^∫^	0.344 (0.033–3.557)	0.371
Leflunomide, *n* (%)	9 (32.1)	13 (39.4)	0.602^∫^	0.479 (0.151–1.517)	0.211
TNF inhibitors, *n* (%)a	3 (10.7)	5 (15.2)	0.715^∫^	0.851 (0.173–4.193)	0.842
Statin, *n* (%)	5 (17.9)	2 (6.1)	0.231^∫^	1.992 (0.379–9.750)	0.430
NSAIDs, *n* (%)	19 (67.9)	26 (78.8)	0.391^∫^	0.470 (0.142–1.544)	0.216

Data are presented as mean ± standard deviation, median [interquartile range], or number (percentage) as appropriate. *CI* confidence interval, *NA* not applicable, *BMI* body mass index, *HDL* high-density lipoprotein, *LDL* low-density lipoprotein, *ESR* erythrocyte sedimentation rate, *CRP* C-reactive protein, *RF* rheumatoid factor, *Anti-CCP antibody* anti-cyclic citrullinated peptide antibody, *DAS28* Disease Activity Score in 28 joints *TNF* tumor necrosis factor, *NSAIDs* non-steroidal anti-inflammatory drugs

^†^Unless otherwise noted, Mann-Whitney *U* test was used

^‡^Logistic regression was used to obtain age-adjusted odds ratios for the presence of diastolic dysfunction

^∫^Chi-square test was performed first and Fisher’s two-tailed exact test was used if warnings were presented

^∫∫^Two-sample *t* test was used

^¶^Indicated antibody positivity

**Fig. 1:**
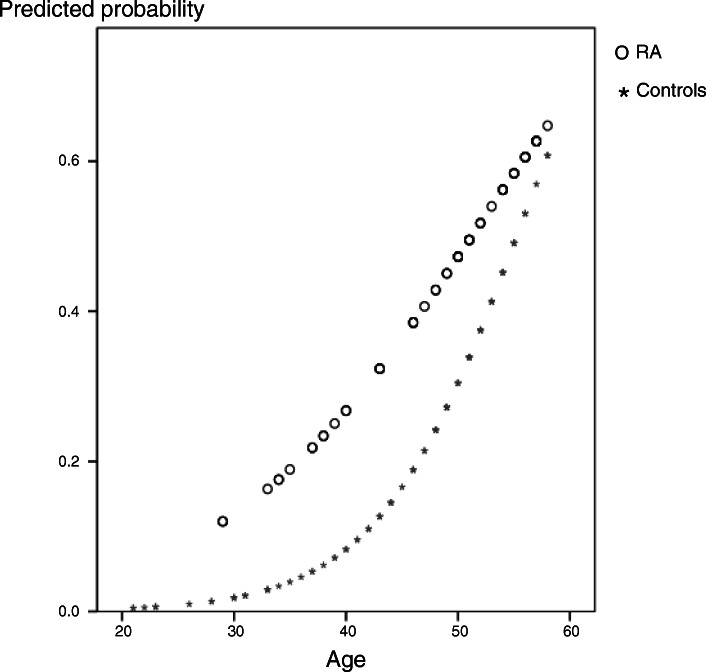
Predicted probability plot of diastolic dysfunction according to age in the study group: patients with rheumatoid arthritis (RA) (o) and controls (*)

The predicted probability of diastolic dysfunction increased with increasing age in both groups. However, although the probability of diastolic dysfunction was not different between the elderly group, RA patients under 50 years of age showed an increased risk of diastolic dysfunction (Fig. 1). In particular, women with RA in the 30- to 49-year age group were over 3.5 times more likely to have diastolic dysfunction than those of similar age in the control group (OR=3.54; 95% CI 1.27 to 9.85).

## Discussion

Patients with RA have a high CVD mortality rate and heart failure is a major contributing factor to CVD. An earlier study showed that patients with RA had twice the risk for development of heart failure as control subjects [1]. Subclinical diastolic dysfunction is known to be a major antecedent risk factor for future development of symptomatic heart failure [17]. In this respect, it is important to investigate diastolic dysfunction in RA patients who are asymptomatic for cardiovascular disease. This study demonstrated that the prevalence of diastolic dysfunction in patients with RA was increased in premenopausal women, and this was associated with inflammation.

Clinical studies have found advanced age, hypertension, and women are associated with increased diastolic dysfunction. Most of the studies have been conducted on old-age patients. In particular, the risk of diastolic dysfunction increases sharply in postmenopausal women. It is known that the protective effect of estrogen against hypertension and ventricular remodeling decreases after menopause [18]. This suggests that it is necessary to investigate separately women by age, since the disease risk varies from menopause. Regarding hypertension, hypertension itself does not only significantly affect the stiffness of blood vessels, but it is also one of the strongest risk factors for diastolic dysfunction. The presence of hypertension implies that structural changes in the heart have already begun. For this reason, we narrowed the inclusion criteria and compared with the general population whether RA affects heart function in premenopausal women without a history of cardiovascular disease.

Comparison of the incidence of diastolic dysfunction is illustrated in Fig. 1. The significant influence that age has on the development of diastolic dysfunction is well known [17]. This study also showed that the predicted probability of diastolic dysfunction increased with age in both groups. Interestingly, the control group had an “S-shaped” pattern showing a rapid increase from the mid-40s, whereas the RA patients showed an almost linear increase from the 30s. RA patients aged 30–50 already have a risk of developing diastolic dysfunction comparable to control subjects in their 60s. This indicates that the risk of diastolic dysfunction in RA patients is accelerated, which is in line with the observation that patients with RA over the age of 60 experience a significantly higher incidence of CHF compared to those without [1]. This increased risk of diastolic dysfunction at a younger age is undoubtedly one of the reasons contributing to heart failure at an older age.

Obesity and dyslipidemia are recognized as important risk factors for diastolic dysfunction [17]. Obesity is associated with ventricular remodeling, which may normalize wall stress while increasing stroke volume to match metabolic demand [19]. A relationship between left ventricle dysfunction and serum lipid levels has also been reported [20]. However, as shown in Table 1, the BMI of RA patients was lower than that of the control population. In addition, although seven more RA patients were taking statins, the patient group showed a more favorable lipid profile than controls. These observations suggest that the increased risk is not explained by traditional cardiovascular risk factors.

A recent systematic review found a consistent association between diastolic dysfunction and the risk of cardiovascular events and death in community-based populations with different risk factors. As well, individuals with diastolic dysfunction showed a 3.53-fold higher risk of cardiac events or death and a 3.13-fold increased risk of mortality [21]. A meta-analysis found there is an increased prevalence of diastolic dysfunction in RA patients. In this study, the prevalence of diastolic dysfunction, LV mass index, and LV filling pressure were higher in RA patients than in the control group. However, systolic tissue velocity was decreased for these patients. Systolic tissue velocity is an early marker of myocardium contraction.

In this study, serum CRP levels were significantly associated with the risk of diastolic dysfunction in RA and this association remained after adjustment for age and other factors. Inflammation promotes endothelial dysfunction, myocardial leukocyte infiltration, and oxidative stress [22]. In vitro studies have also revealed that inflammation is involved in cardiac remodeling, resulting in diastolic dysfunction. Indeed, HF patients have high levels of inflammatory cytokines such as TNF-α, IL-6, and IL-1ß. These proinflammatory cytokines are correlated with a deterioration of functional and cardiac performance as well as prognostic markers of HF [23]. Furthermore, another study demonstrated increased inflammatory cytokines and diastolic heart failure in patients with RA [10]. Schwartz reported histopathological features such as non-specific myocarditis, myocardial granulomatous lesions, secondary amyloidosis, and diffuse fibrosis in RA [24]. Taken together, the data suggest that inflammation leads to myocardial dysfunction in RA.

Several studies have reported a significant inverse relationship between the E/A ratio and the disease duration [10, 25, 26]. However, the clinical relevance of diastolic dysfunction and its possible relationship to disease duration has not been yet clarified. The study by Montecucco et al. [27] found no association between diastolic dysfunction and disease duration. In this study, we also could not find a significant association between these two variables. Disease duration is a comprehensive variable that implies the accumulation of time and chronic inflammation. But it also includes the period of well-controlled inflammation. Furthermore, it is difficult to estimate the disease duration accurately because the disease duration is calculated according to the patient’s memory and hospital visit time. This aspect may seem to lead to inconsistent results.

This study has several limitations. First, it did not include patients with hypertension. In the process of studying the prevalence of diastolic heart failure in asymptomatic patients, the age of the enrolled subjects was relatively young. There were very few patients with hypertension, and therefore not enough data for statistical analysis. In the future, we plan to conduct studies in which hypertension patients are stratified and analyzed. Second, this study was conducted in women. According to epidemiological data, the prevalence of RA varies depending on both age and gender. In premenopausal women, the difference in incidence ratio by gender is up to 4.8 times, but for those over 60 it is less than doubled to 1.8. The reason for this distribution is not yet fully explained, but clearly, there are differences between women and men, and consequently, caution should be exercised in generalizing across different RA patients.

In summary, premenopausal RA women have a three-fold increased risk of diastolic dysfunction compared to control subjects. Inflammation was related to subclinical myocardial involvement. This study provides evidence in premenopausal RA women that early screening of myocardial function may provide an opportunity for preventing future cardiovascular disease.

## Supplementary Information

**Additional file 1:.** Supplementary Table 1. Multivariate logistic regression analysis

## Data Availability

The datasets used and/or analyzed in the current study are available from the corresponding author on reasonable request.

## Abbreviations

Anti-CCP: Anti-cyclic citrullinated peptide
BMI: Body mass index
CVD: Cardiovascular disease
CI: Confidence interval
CRP: C-reactive protein
DD: Diastolic dysfunction
DAS28: Disease Activity Score 28 Joints
ESR: Erythrocyte sedimentation rate
HF: Heart failure
HDL: High-density lipoprotein
IL: Interleukin
LV: Left ventricular
NSAIDs: Non-steroidal anti-inflammatory drugs
RA: Rheumatoid arthritis
RF: Rheumatoid factor
SD: Standard deviation
TNF-α: Tumor necrosis factor-alpha
