# Supplementary material for: Accelerated diastolic dysfunction in premenopausal women with rheumatoid arthritis
Source: Arthritis Res Ther. 2021 Sep 24;23:247. doi: 10.1186/s13075-021-02629-1 (PMC8461933; doi:10.1186/s13075-021-02629-1)
Supplement: Supplementary file 1 — Additional file 1:. Supplementary Table 1. Multivariate logistic regression analysis [file 13075_2021_2629_MOESM1_ESM.docx]

**Supplementary information**

**Accelerated Diastolic Dysfunction in Premenopausal Women**

**with Rheumatoid Arthritis**

GeeHee Kim M.D.,Ph.D.^1^, Yune-Jung Park M.D.,Ph.D.^2^

^1^Division of Cardiology, Department of Internal Medicine, St. Vincent’s Hospital, College of Medicine, The Catholic University of Korea, Suwon, Korea; ^2^Division of Rheumatology, Department of Internal Medicine, St. Vincent’s Hospital, College of Medicine, The Catholic University of Korea, Suwon, Korea

Correspondence and reprint requests to:

Dr. Yune-Jung Park, ^2^Division of Rheumatology, Department of Internal Medicine, St. Vincent’s Hospital, College of Medicine, The Catholic University of Korea, Suwon, Korea.

E-mail: jwas@catholic.ac.kr

Tel: 82-31-881-8918

Supplementary Table 1. Multivariate logistic regression analysis

|  | Multivariate logistic regression model | | | |
| --- | --- | --- | --- | --- |
|  | OR | P-value | lcl | ucl |
| Age | 1.11 | 0.035 | 1.01 | 1.24 |
| Disease duration | 0.89 | 0.067 | 0.67 | 0.99 |
| Rheumatoid factor positivity | 0.99 | 0.072 | 0.99 | 1.00 |
| C-reactive protein levels | 2.75 | 0.022 | 1.26 | 7.45 |

Multivariate models included following covariates; age, systolic blood pressure, diastolic blood pressure, body mass index, presence of diabetes mellitus, disease duration of rheumatoid arthritis, rheumatoid factor positivity, and anti-cyclic citrullinated protein antibodypositivity. OR; odds ratio, lcl; lower control limit, and ucl; upper control limit.
